# Accelerated cystogenesis by dietary protein load is dependent on, but not initiated by kidney macrophages

**DOI:** 10.3389/fmed.2023.1173674

**Published:** 2023-07-19

**Authors:** Randee Sedaka, Jifeng Huang, Shinobu Yamaguchi, Caleb Lovelady, Jung-Shan Hsu, Sejal Shinde, Malgorzata Kasztan, David K. Crossman, Takamitsu Saigusa

**Affiliations:** ^1^Section of Cardio-Renal Physiology and Medicine, Division of Nephrology, Department of Medicine, University of Alabama at Birmingham, Birmingham, AL, United States; ^2^Division of Pediatric Hematology/Oncology, Department of Pediatrics, University of Alabama at Birmingham, Birmingham, AL, United States; ^3^Department of Genetics, University of Alabama at Birmingham, Birmingham, AL, United States

**Keywords:** polycystic kidney disease, high protein, glutamine, renal hypertophy, macrophage

## Abstract

**Background:**

Disease severity of autosomal dominant polycystic kidney disease (ADPKD) is influenced by diet. Dietary protein, a recognized cyst-accelerating factor, is catabolized into amino acids (AA) and delivered to the kidney leading to renal hypertrophy. Injury-induced hypertrophic signaling in ADPKD results in increased macrophage (MФ) activation and inflammation followed by cyst growth. We hypothesize that the cystogenesis-prompting effects of HP diet are caused by increased delivery of specific AA to the kidney, ultimately stimulating MФs to promote cyst progression.

**Methods:**

*Pkd1*^flox/flox^ mice with and without Cre (CAGG-ER) were given tamoxifen to induce global gene deletion (*Pkd1*KO). *Pkd1*KO mice were fed either a low (LP; 6%), normal (NP; 18%), or high (HP; 60%) protein diet for 1 week (early) or 6 weeks (chronic). Mice were then euthanized and tissues were used for histology, immunofluorescence and various biochemical assays. One week fed kidney tissue was cell sorted to isolate tubular epithelial cells for RNA sequencing.

**Results:**

Chronic dietary protein load in *Pkd1KO* mice increased kidney weight, number of kidney infiltrating and resident MФs, chemokines, cytokines and cystic index compared to LP diet fed mice. Accelerated cyst growth induced by chronic HP were attenuated by liposomal clodronate-mediated MФ depletion. Early HP diet fed *Pkd1*KO mice had larger cystic kidneys compared to NP or LP fed counterparts, but without increases in the number of kidney MФs, cytokines, or markers of tubular injury. RNA sequencing of tubular epithelial cells in HP compared to NP or LP diet group revealed increased expression of sodium-glutamine transporter *Snat3*, chloride channel *Clcnka*, and gluconeogenesis marker *Pepck1*, accompanied by increased excretion of urinary ammonia, a byproduct of glutamine. Early glutamine supplementation in *Pkd1*KO mice lead to kidney hypertrophy.

**Conclusion:**

Chronic dietary protein load-induced renal hypertrophy and accelerated cyst growth in *Pkd1*KO mice is dependent on both infiltrating and resident MФ recruitment and subsequent inflammatory response. Early cyst expansion by HP diet, however, is relient on increased delivery of glutamine to kidney epithelial cells, driving downstream metabolic changes prior to inflammatory provocation.

## Introduction

Autosomal dominant polycystic kidney disease (ADPKD) is the most common genetic form of kidney disease, with approximately 50% of patients developing end-stage kidney disease (ESKD) by mid-life. In most cases, ADPKD results from germline mutations in the *PKD1* or *PKD2* genes, both of which are characterized by the formation of fluid-filled cysts along the kidney tubular epithelium concurrent with kidney enlargement. Cysts appear earlier and grow more rapidly, however, in *PKD1*-null cells (1). Despite this genetic commonality, disease progression is highly variable, even amongst family members that share identical mutations (2). Moreover, there are a significant subset of ADPKD patients who develop severely accelerated cystic disease leading to ESKD much earlier in life (3–5).

This variability may be due, in part, to the effects of non-genetic factors that promote cyst growth in PKD (6–9). Compelling evidence suggests that compensatory renal hypertrophy triggered by unilateral nephrectomy (9, 10) or high protein intake (11–13) can exacerbate cyst progression in rodents. The hypertrophic kidney response involves kidney hyperfiltration (14) and increased tubular delivery of amino acids and humoral factors (15). However, the mechanism of how compensatory renal hypertrophy superimposes accelerated cystogenesis in ADPKD is unknown.

Recruitment of MФs and inflammatory factors in the kidney plays an essential role in promoting cystogenesis in murine models of PKD (16–19). Additionally, determining the origin of said macrophages, between bone marrow–derived infiltrating (CD11b^hi^, F4/80^lo^) and tissue-resident (CD11b^lo^, F4/80 ^hi^), helps to not only contexualize the disease state, but provides insights into the ontology and polarization capacity of the MФ population (20, 21). We previously reported that unilateral nephrectomy increases the number of kidney resident macrophages (MФ) and pro-inflammatory cytokines, accelerating cyst progression in mice lacking *Pkd1* (*Pkd1*KO) (22). Therefore, we hypothesize that dietary protein load increases kidney MФs and accelerates cyst growth in *Pkd1KO* mice similarly to the unilateral nephrectomy model.

In this study, we found that chronic dietary protein load increases kidney MФ numbers and cytokine expression in *Pkd1KO* mice, yielding accelerated cystogenesis. Renal hypertrophy, cyst growth, and inflammation induced by high protein were blocked by macrophage depletion. During the early stages of cyst progression via high protein load, kidney epithelial cells upregulate expression of gluconeogenesis marker *Pepck1*, chloride channel *Clcnka*, and sodium-coupled glutamine transporter *Slc38a3*, prior to the rise in kidney MФs. Kidney hypertrophy at this early stage was relient on glutamine. Thus, early metabolic alterations in kidney epithelial cells exposed to a high protein load precede immune involvement in initiating cyst growth and hypertrophy.

## Methods

### Animals

Experimental protocols were approved by the Institutional Animal Care and Use Committee at the University of Alabama at Birmingham and performed in accordance with the National Institutes of Health Guide for the Care and Use of Laboratory Animals. Conditional *Pkd1* knockout (*Pkd1KO*) mice were generated by crossbreeding female *Pkd1*-floxed mice (23) with male *Pkd1*-floxed mice containing a tamoxifen inducible systemic Cre (CAGG-CreER) (24). Genotyping was performed by PCR using previously described primer sequences (23), denoting mice lacking cre as “flox” controls. Male and female mice were equally included in each study. Mice were maintained under constant temperature and humidity conditions, a 12:12 h light–dark cycle, and water *ad libitum*. Flox and *Pkd1KO* mice (5–6 weeks old) received an intraperitoneal (IP) injection of Tamoxifen (T5648, Millipore Sigma, Burlington, MA; 9 mg/40 g) dissolved in corn oil every other day for a total of three doses. Kidney and spleen tissue were harvested under constant isoflurane inhalation followed by thoracotomy and either frozen at -80°C for biochemical analyses or immersed in respective buffers for flow cytometry or staining. For all data presented, each datapoint represents one mouse.

### Dietary intervention

**Figure 1:**
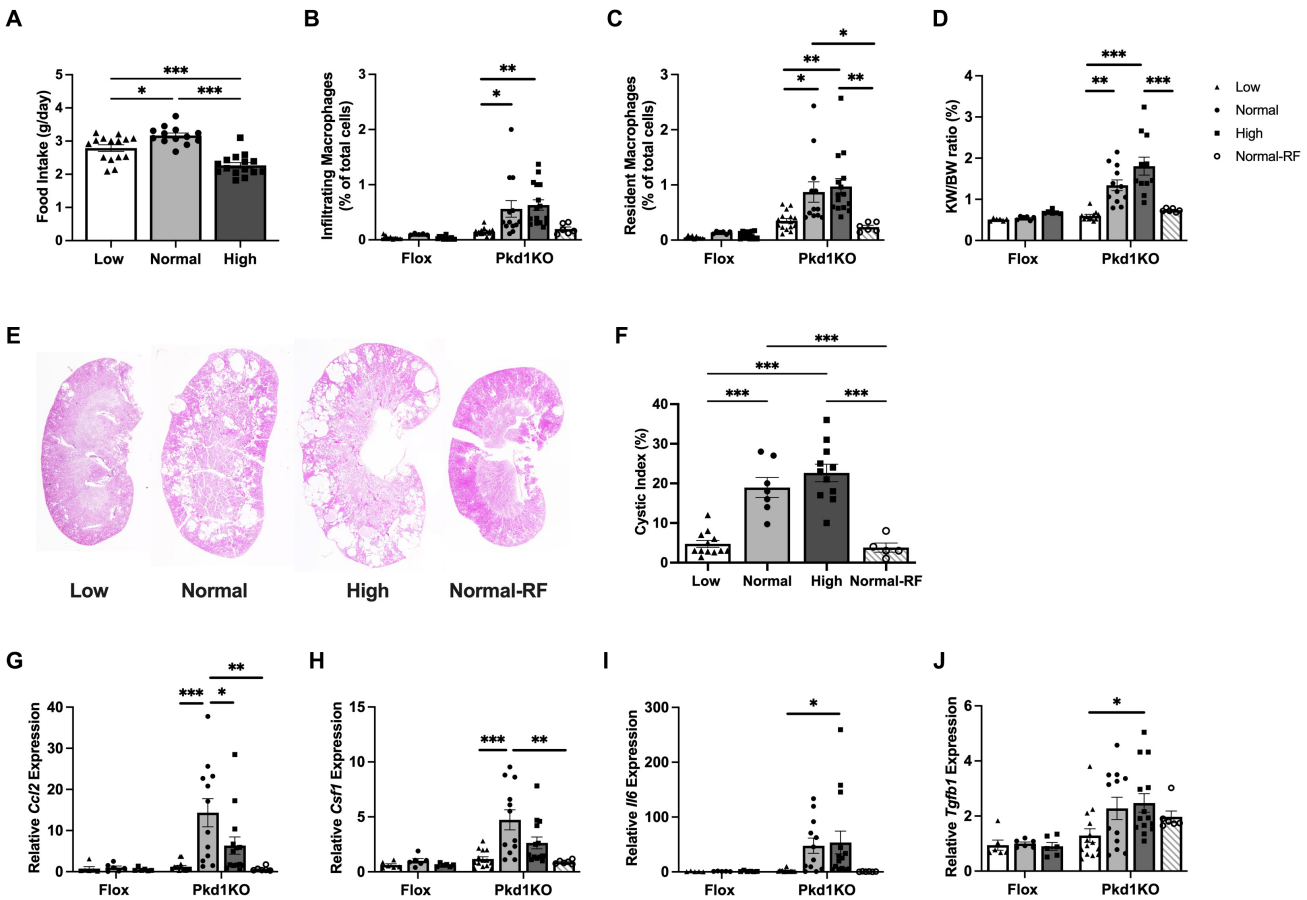
*Pkd1KO* mice fed a chronic high protein load have increased kidney expression of MФ chemoattractants and MФ numbers. **(A)** Average daily food intake during 6 week *ad libitum* dietary intervention. *Pkd1KO* mouse ate more NP versus LP or HP diet. **(B,C)** Number of infiltrating and resident MФs, shown as a percentage of total cells amongst diets and genotypes. The number of infiltrating MФs in LP and resident MФs in LP and Normal-RF fed *Pkd1KO* mice were lower compared to NP or HP fed mice. **(D)** Kidney weight to body weight ratio (KW/BW). *Pkd1KO* mice fed a HP diet had elevated KW/BW ratio compared to those fed a Normal-RF or LP diet. There was an overall significant effect of genotype and diet in **(B–D)**. **(E,F)** Representative *Pkd1KO* kidney histology and cystic index. Cystic index was lower in LP and Normal-RF compared to NP or HP fed *Pkd1KO* mice. **(G–J)** Kidney gene expression of *Ccl2, Csf1, Il6 and Tgfb1*. No differences were observed in flox mice. *Ccl2* and *Csf1* expression was lower in both LP and Normal-RF fed *Pkd1KO* mice compared to NP diet group. *Il6* and *Tgfb1* expression was lower only in LP compared to HP fed *Pkd1KO* mice. Gene expression is relative to flox mice fed a NP diet. Results of a two-way ANOVA or Ordinary one-way ANOVA with Tukey’s multiple comparisons test reported. *p < 0.05, **p < 0.01, ***p < 0.001.

Two weeks post-tamoxifen injection, mice were individually housed and randomly assigned to consume a low (6%; TD.90016), normal (18%; TD.96180) or high (60%; TD.6220) protein diet for 6 weeks *ad libitum*. All diets were formulated by Envigo (Indianapolis, IN) to be isocaloric (3.7 kcal/g). Nutritional and amino acid composition of each diet are shown in Supplementary Tables S1, S2, respectively. Based on the overall average weekly food intake (Figure 1A), an additional group of KO mice were restricted to 2.8 g/day of normal protein diet (Normal-RF) for 6 weeks. In a separate cohort, mice placed on all three diets were food restricted (2.8 g/day) for 1 week.

### Flow cytometry

The gating strategy for selecting infiltrating (CD11b^hi^, F4/80^lo^) and tissue-resident (CD11b^lo^, F4/80^hi^) macrophages was previously described (25). Briefly, after cardiac perfusion with phosphate buffer saline (PBS; MT21040CV, Fisher Scientific, Waltham, MA), kidneys were minced and digested in collagenase type I containing buffer. Fragments of kidney were passed through 70 *μ*m mesh, yielding a single-cell suspension. After red blood cell lysis, cells were blocked, stained with primary antibodies (Supplementary Table S3), fixed in 2% paraformaldehyde and resuspended in PBS. Spleens were utilized for single color staining controls. Cells were analyzed on a BD LSRII flow cytometer and data analysis was performed using FlowJo version 10 software.

### Quantitative real-time PCR

Total RNA was isolated from kidney tissue using TRIzol Reagent (15-596-018, Fisher Scientific) according to the manufacturer’s instructions. RNA (1.0 μg) was reverse transcribed using Bio-Rad’s iScript cDNA Synthesis Kit (1708891, Hercules, CA) or Applied Biosystems’ High-Capacity cDNA Reverse Transcription Kit (4368814, Fisher Scientific). Relative expression of individual genes was determined using SsoAdvanced Universal SYBR Green Supermix (1725271, Bio-Rad) and primers synthesized by Integrated DNA Technologies (Supplementary Table S4; Coralville, IA) or TaqMan Gene Expression Master Mix (4369514, Fisher Scientific) and Taqman probes for *Ccl2* (Mm00441242), *Csf1* (Mm00432686), *Il-6* (Mm00446190), *Tgfb1* (Mm01188201), *Cmyc* (Mm01192721), and *Gapdh* (4352339E, Fisher Scientific). qRT-PCR was conducted on a CFX96 Touch Real-Time PCR Detection System (Bio-Rad), and data were normalized to *Gapdh*.

### Cyst quantification

Sections (5 *μ*m) were cut from paraffin-embedded kidneys and stained with hematoxylin–eosin (H&E). Whole kidney images were captured at 4x magnification using a Keyence BZ-X710 (Itasca, IL) microscope. In Image J, images were split from RGB to individual color channels, then the green channel was converted into B&W to analyze particles. Tubular lumen diameters were measured and manually evaluated to be dilated tubules/cysts if over 0.50 mm. We then took the sum of the thresholded “cystic” area and divided it by the total kidney area to calculate cystic index. Mean cystic area relative to total kidney section area are shown as a percentage. A technician blinded to the treatment groups performed the quantification.

### Macrophage depletion

Three weeks after tamoxifen injection, *Pkd1KO* mice were placed on a high protein diet and IP injected with either Liposomal Clodronate (C-020, Liposoma BV, Amsterdam, Netherlands; 50 mg/kg) or PBS twice weekly for a total of 4 weeks. Mice were euthanized 2 days after the last dose of Clodronate or PBS.

### Blood urea nitrogen assay

Blood was removed by cardiac puncture while mice were anesthetized under constant isoflurane inhalation and centrifuged at 2,000 g for 10 min to collect plasma. Blood urea nitrogen (BUN) was measured in diluted plasma utilizing a Urea Nitrogen Colorimetric Detection Kit (K024-H1, Arbor Assays, Ann Arbor, MI) following the manufacturer’s protocol.

### RNA sequencing

Kidneys from *Pkd1KO* mice fed a low, normal, or high protein diet (2.8 g/day) for 1 week were collected, as in the flow cytometry protocol, and flow sorted to collect an epithelial cell population. In short, kidney single-cell suspensions were stained with markers to detect infiltrating and resident macrophages (CD11b and F4/80), as well as proximal tubule (Fluorescein labeled *Lotus tetragonolobus* lectin (LTL); FL-1321, Vector Laboratories, Newark, CA) and collecting duct (Rhodamine labeled *Dolichos biflorus* agglutinin (DBA); RL-1032, Vector Laboratories) epithelium as previously described (22). Cells were sorted into individual tubes using a BD Biosciences FACSAria III. RNA was isolated using TRIzol Reagent. Approximately 100–200 ng of total RNA was used to prepare cDNA libraries with the Stranded mRNA-Seq Library Preparation Kit (Illumina) according to the manufacturer’s protocol. High-throughput sequencing (HTS) was performed using an Illumina HiSeq 2,500 (Genewiz, South Plainfield, NJ). Sequence reads in fastq format were trimmed using TrimGalore (version 0.6.7) to remove any primer adapter contamination. RSEM (version 1.3.3) was used to align the trimmed RNA-Seq fastq reads to the mouse reference genome from Gencode (GrCh39 Release M26). The raw sequencing and processed data have been submitted to Gene Expression Omnibus with accession number (GSE# 225495). Analyses were performed on an *n* = 2–3 per diet, with each “*n*” represented by a pooled sample from 2–3 mice. Due to insufficient RNA in one sample, gene set enrichment pathway analysis was not conducted.

### Western blot

*Pkd1KO* kidney tissue samples were homogenized in ice-cold T-PER Tissue Protein Extraction Reagent (78510, Fisher Scientific) in the presence of protease (Halt Protease Inhibitor Cocktail; 87785, Fisher Scientific) and phosphatase inhibitors (Halt Phosphatase Inhibitor Cocktail; 78420, Fisher Scientific). Homogenates were centrifuged at 10,000 rpm for 5 min to remove cellular debris and supernatant protein concentrations were determined by Bradford assay (Quick Start Bradford 1x Dye Reagent; 5000205, Bio-Rad), then boiled with 4x Laemmli sample buffer (1610747, Bio-Rad) and 5% β-mercaptoethanol for 5 min at 90°C. Proteins were separated by SDS-PAGE, transferred to Immobilon-FL polyvinylidene difluoride (PVDF) membrane (IPFL00010, Millipore Sigma), blocked with 5% milk for 1 h at room temperature, and incubated overnight shaking at 4°C with corresponding primary antibodies (Supplementary Table S3) in TBS with 0.1% Tween 20 (TBST). After incubation with secondary antibody (Goat anti-Mouse IgG, DyLight 800 [SA5-10176] or Goat anti-Rabbit IgG, DyLight 680 [35569]; Fisher Scientific) for 1 h at room temperature, the membrane was washed with TBST and scanned using the Odyssey CLx Infrared Imaging System (LI-COR Biosciences). Densitometry was performed using LI-COR Image Studio Lite. Equal protein loading was verified by β-actin staining and shown in each representative figure.

### Immunofluorescent staining

Kidneys were snap frozen, sectioned, and stained as previously described (22). Briefly, slides were air-dried at room temperature for 30 min and fixed with 4% PFA, permeabilized with 0.2% TritonX, then blocked with 1% Goat Serum (ab7481). Sections were incubated with unconjugated primary antibody SNAT3 (Supplementary Table S3) for 1 h, conjugated fluorescence primary antibody (LTL: Vector FL-1321-2) for 20 min, followed by Goat anti-Mouse Alexa Fluor 594 (1:1000; A-11005, Invitrogen) secondary antibody for 1 h in RT, and Hoechst 33342 nuclear stain (H3570, Fisher Scientific) for 5 min. Fluorescent images were captured at 10x magnification using a Keyence BZ-X710 Microscope. The scale bar is equivalent to 100 *μ*m.

### Ammonia assay

Ammonia was measured in random spot urine and kidney homogenates (20 mg/0.5 mL deionized water) using Cell Biolabs, Inc. colorimetric Ammonia Assay (MET-5086, San Diego, CA) according to the manufacturer’s protocol. Urinary ammonia was normalized to creatinine utilizing the Creatinine Colorimetric Assay Kit (500701, Cayman Chemicals, Ann Arbor, Michigan).

### Glutamine studies

Two weeks after tamoxifen injection, *Pkd1KO* mice were placed on a high protein diet and given an oral gavage (OG) of CB-839 (HY-12248, MedChemExpress, Monmouth Junction, NJ; 100 mg/kg) or vehicle daily for 1 week. Vehicle contained 25% (w/v) hydroxypropyl-β-cyclodextrin (HY-101103, MedChemExpress) in 10 mM sodium citrate (pH 2.0; C7254, Millipore Sigma). In a separate cohort, *Pkd1KO* mice fed a normal protein diet were gavaged with glutamine (G8540, Millipore Sigma; 5 g/kg) or vehicle (1X PBS) daily for 1 week. For both studies, mice were euthanized 1 day after the last dose.

### Statistical analyses

Data are presented as means ± SEM. Differences between group means were analyzed using GraphPad Prism 9 (La Jolla, CA) by two-tailed, unpaired Student’s *t* test, Ordinary one-way ANOVA with Tukey’s multiple comparisons *post hoc* test or two-way ANOVA with Tukey’s multiple comparisons *post hoc* test, as noted. *p* < 0.05 denoted statistically significant.

## Results

### Dietary protein restriction decreases kidney MФ recruitment and expansion, slowing cyst growth in *Pkd1KO* mice

To determine the involvement of innate immunity in protein-accelerated cystogenesis, flox and *Pkd1KO* mice were fed a low (LP), normal (NP), or high (HP) protein diet for 6 weeks. Average daily food intake of NP fed *Pkd1KO* mice was higher than counterparts fed LP or HP (Figure 1A). As a result, an additional group of *Pkd1KO* mice were placed on a restricted NP diet for 6 weeks to approximately match the average caloric intake of the LP and HP fed mice. Restricted NP fed *Pkd1KO* mice had lower body weights compared to those fed *ad libitum* (Supplementary Figure S1A), even though food intake to body weight ratio was comparable to *ad lib* fed NP mice (Supplementary Figure S1B).

*Pkd1KO* mice fed a LP diet had fewer infiltrating and resident MФs (Figures 1B,C), a decreased kidney weight to body weight (KW/BW) ratio (Figure 1D), and a five-fold lower cystic index (Figures 1E,F) compared to NP or HP diet counterparts. Similarly, restricting NP resulted in fewer resident MФs, a decreased KW/BW ratio, and five-fold fewer cysts/dilated tubules compared to higher protein load diets (Figures 1C–F). Kidney expression of MФ-recruiting chemokine *Ccl2* and MФ-expanding cytokine *Csf1* was nearly fifteen-and five-fold lower, respectively, in LP or restricted NP diet versus *ad libitum* NP fed *Pkd1KO* mice (Figures 1G,H). Pro-inflammatory cytokine *Il6* (Figure 1I) and pro-fibrotic cytokine *Tgfb1* (Figure 1J) were suppressed in LP compared to HP fed *Pkd1KO* mice by approximately twenty- and two-fold, respectively. Flox control mice had significantly lower numbers of kidney MФs and expression of associated chemokines and cytokines compared to *Pkd1KO* mice, irrespective of diet.

### MФ depletion slows cyst growth during high dietary protein load in *Pkd1KO* mice

**Figure 2:**
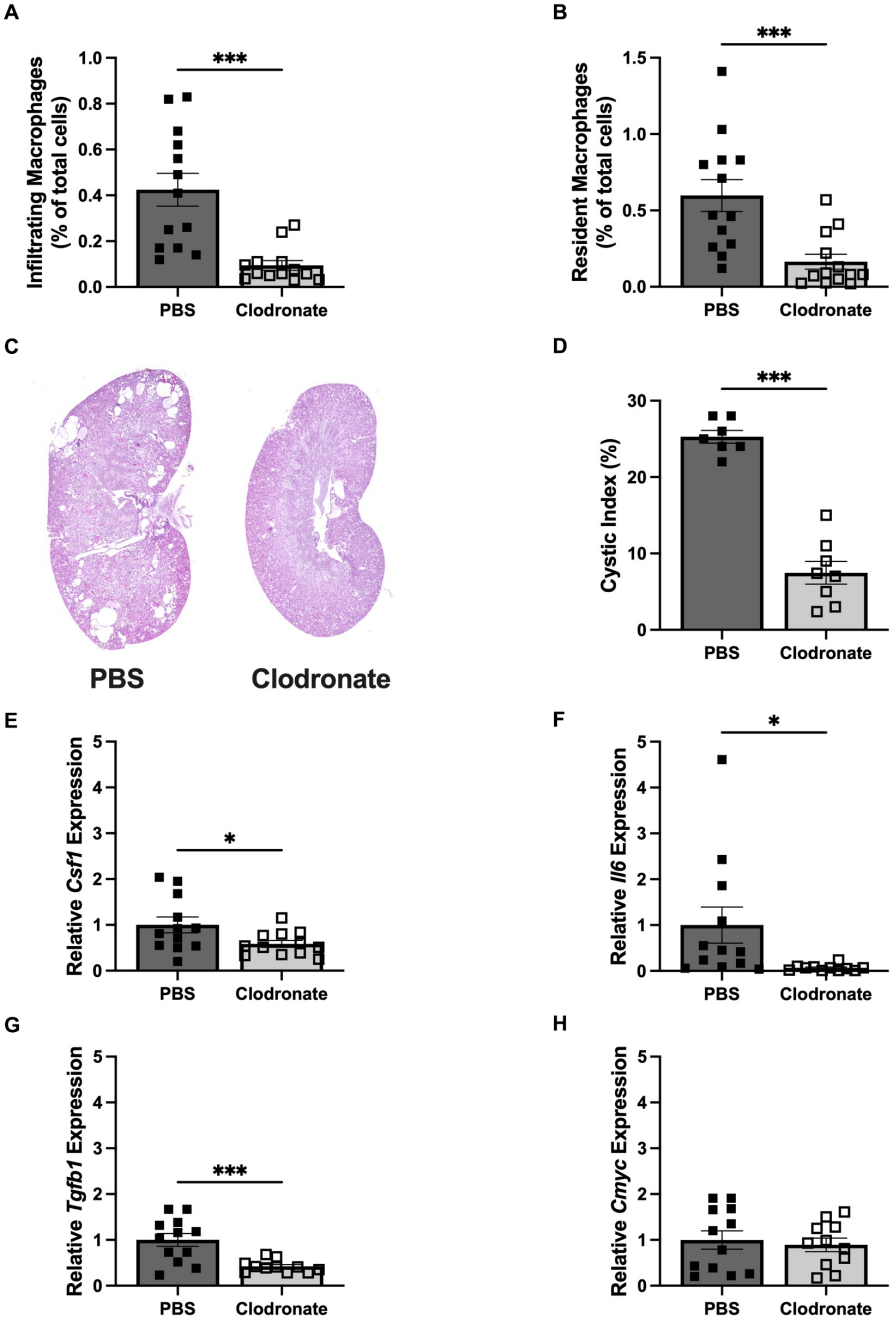
MФ depletion via liposomal clodronate slows cyst progression in HP loaded *Pkd1KO* mice. **(A,B)** Number of infiltrating and resident MФs, shown as a percentage of total cells amongst treatment groups. Twice weekly clodronate (50 mg/kg, IP) treatment suppressed both infiltrating and resident MФs compared to PBS treatment. **(C,D)** Representative *Pkd1KO* kidney histology and cystic index. Cyst growth was blunted by clodronate treatment. **(E–H)** Kidney gene expression of *Csf1, Il6, Tgfb1* and *Cmyc*. Expression of *Csf1, Il6* and *Tgfb1* were significantly lower in clodronate treated mice, without changes in *Cmyc* expression. Gene expression is relative to PBS-treated, HP-fed *Pkd1KO* mice. Results of unpaired, Student’s *t*-test reported. *p < 0.05, ***p < 0.001.

Depleting macrophages with liposomal clodronate was previously shown to slow cyst growth in rodent models of PKD (16, 18). Therefore, we tested whether MФ depletion slows accelerated cyst growth triggered by a dietary protein load. *Pkd1KO* mice treated with clodronate had fewer numbers of both infiltrating and resident MФs compared to counterparts given PBS (Figures 2A,B). Cyst progression was reduced three-fold in clodronate compared to PBS treated mice (Figures 2C,D). Likewise, MФ depletion resulted in lower expression of *Csf1*, *Il6* (10-fold) and *Tgfb1*, with no differences in proliferation marker *Cmyc* (Figures 2E–H).

### Early kidney cyst growth from dietary protein load precedes the increase in kidney MФs and inflammation

**Figure 3:**
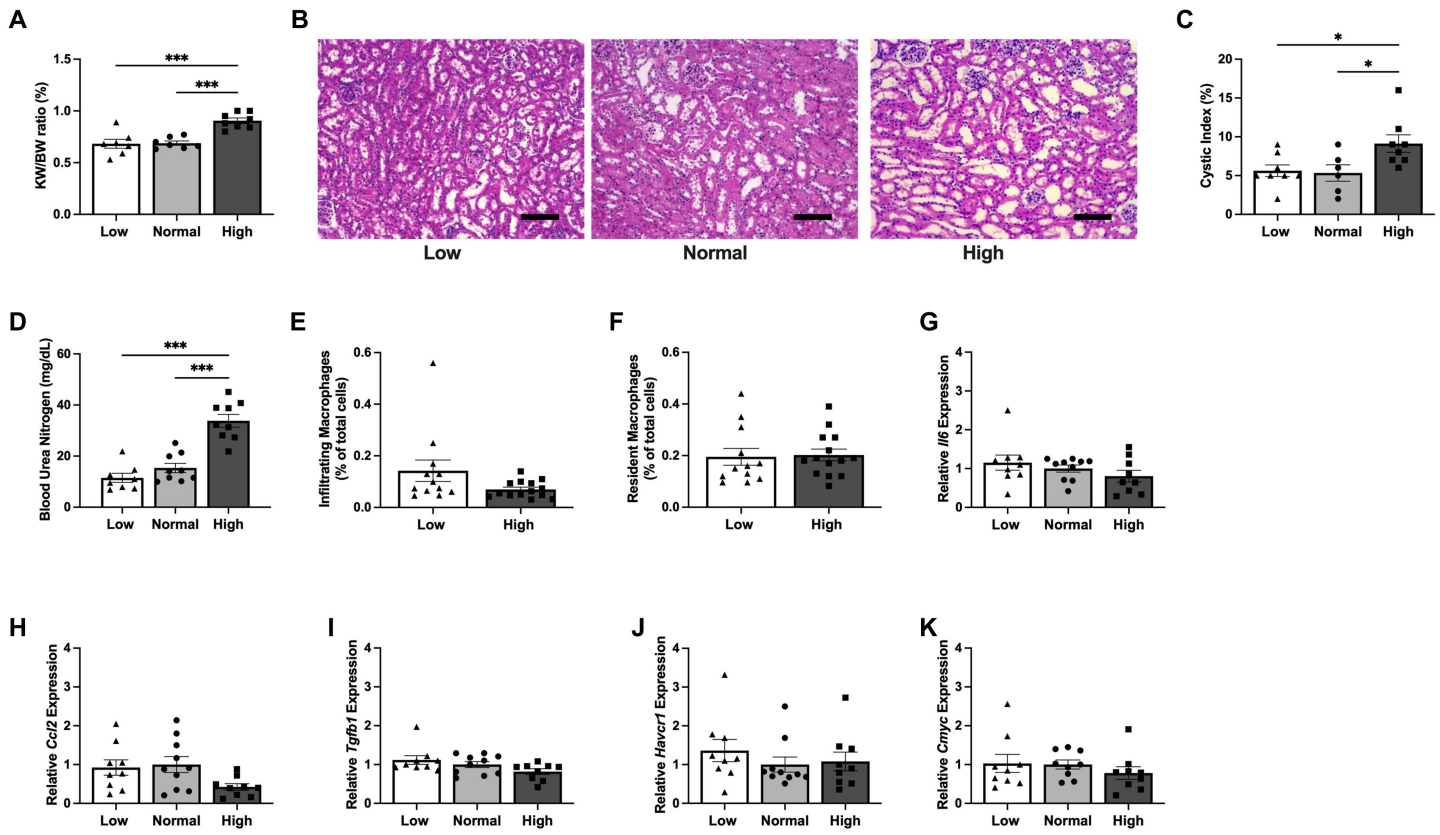
Kidney cyst expansion initiates prior to the increase in inflammatory response during early dietary protein load in *Pkd1KO* mice. **(A–C)** KW/BW ratio, representative kidney histology, and kidney cystic index from *Pkd1KO* mice fed a low, normal, or high protein diet for 1 week. KW/BW ratio and cystic index were higher in HP-fed compared to LP- or NP-fed counterparts (scale bar: 100 *μ*m, 10x magnification). **(D)** Plasma blood urea nitrogen (BUN). HP-fed mice had elevated BUN verses LP- or NP-fed mice. **(E,F)** Number of infiltrating and resident MФs, shown as a percentage of total cells amongst diets. No differences were observed between diets in the number of either MФ. **(G–K)** Kidney gene expression of *Il6, Ccl2, Tgfb1, Havcr1* and *Cmyc*. There were no gene expression differences of cytokines, chemokines, or injury markers amongst mice fed LP, NP, or HP diets. Gene expression is relative to NP-fed *Pkd1KO* mice. Results of an Ordinary one-way ANOVA with Tukey’s multiple comparisons or unpaired, Student’s *t*-test reported. *p < 0.05, ***p < 0.001.

Due to the observed differences in food intake and body weight during the above 6 week study, an additional cohort of *Pkd1KO* mice were fed either a LP, NP, or HP diet restricted to the same amount of food per day for 1 week total. There were no differences in BW, but the KW/BW ratio was higher in *Pkd1KO* mice fed a HP diet compared to LP and NP diet counterparts (Figure 3A). Although predominantly driven by dilated tubules and small cysts, the cystic index was already doubled in HP compared to LP or NP fed mice (Figures 3B,C). Blood urea nitrogen was also increased in the HP diet mice compared to lower protein loads (Figure 3D). In spite of these changes, there were no differences in the number of kidney infiltrating or resident MФs between diets (Figures 3E,F). The expression of *Il6, Ccl2*, *Tgfb1*, proximal tubular injury marker *Havcr1*, and *Cmyc* were similar amongst all diets (Figures 3G–K).

### Dietary protein load increases expression of metabolic markers in kidney tubular epithelial cells

**Figure 4:**
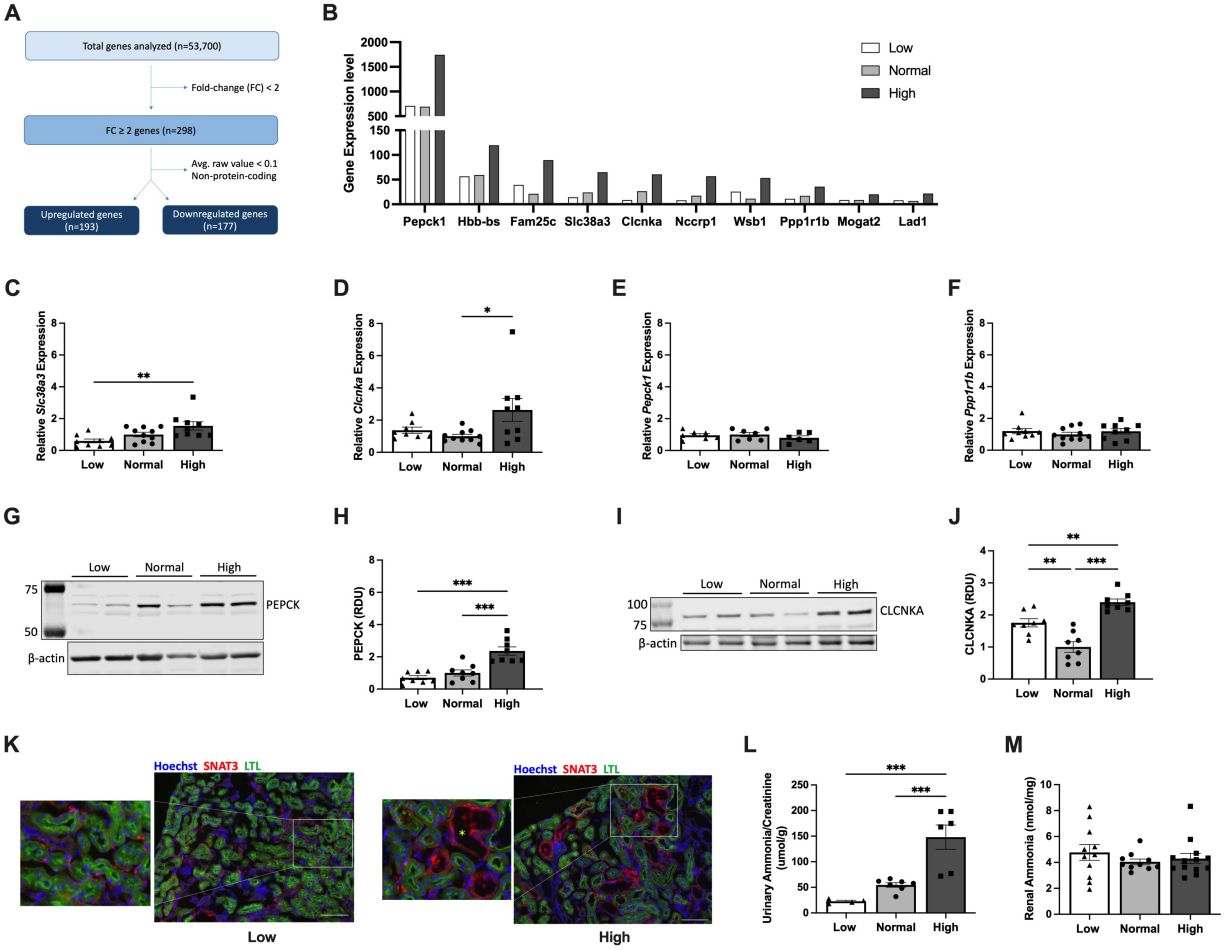
Early dietary protein load upregulates expression of *Slc38a3*, *Pepck1* and *Clcnka* in kidney tubular epithelial cells. **(A)** Flow chart of RNA sequencing analysis using flow-sorted kidney epithelial cells from *Pkd1KO* mice fed a low, normal, or high protein diet for 1 week. **(B)** Top 10 upregulated genes in kidney epithelial cells from HP compared to LP or NP diet fed *Pkd1KO* mice. **(C–F)** Kidney gene expression of *Slc38a3, Clcnka, Pepck1*, and *Ppp1r1b*. Gene expression of *Slc38a3* and *Clcnka* was higher in HP-fed mice compared to LP- or NP- fed mice, respectively. There were no differences in gene expression of *Pepck1* or *Ppp1r1b*. **(G–J)** Representative Western blots and densitometric quantification of PEPCK and CLCNKA. Expression of both proteins is elevated in *Pkd1KO* mice fed a HP versus a LP or NP diet. Gene expression and protein abundance is relative to NP-fed *Pkd1KO* mice. **(K)** Representative immunofluorescent images of kidneys from LP- and HP-fed *Pkd1KO* mice stained for SNAT3 (red), LTL (green), and Hoechst (blue). SNAT3 localizes to the basolateral membrane of both proximal and distal tubules in LP- and HP-fed mice, with prominent expression in dilated distal tubules in HP-fed mice (scale bar: 100 *μ*m, 10x magnification). Yellow asterisk denotes dilated tubule. **(L,M)** Ammonia concentration in urine and kidney tissues from *Pkd1KO* mice fed a LP, NP, or HP diet. Urinary NH3, normalized to urinary creatinine, was higher in HP-fed mice compared to LP or NP counterparts. Diet did not alter kidney NH3 levels. Results of an Ordinary one-way ANOVA with Tukey’s multiple comparisons test reported. *p < 0.05, **p < 0.01, ***p < 0.001.

To investigate the early contributors of protein-accelerated cytogenesis, kidneys from *Pkd1KO* mice fed a LP, NP or HP diet (2.8 g/day) for 1 week were cell sorted to isolate tubular epithelial cells. RNA sequencing analysis yielded 193 genes that were commonly increased and 177 genes that were commonly decreased in HP compared to LP or NP fed mice (Figure 4A and Supplementary Figures S2A,B). The top upregulated gene was phosphoenolpyruvate carboxykinase *(Pepck1)*, a cytosolic enzyme essential for gluconeogenesis (Figure 4B). Confirmatory RT-PCR for several of the top 10 upregulated genes relevant to tubular epithelial metabolism was performed, finding increased expression of sodium-glutamine transporter *Slc38a3* (26) and voltage-gated chloride channel *Clcnka* (27) in the HP compared to NP or LP fed mice (Figures 4C,D), but no differences in *Pepck1* or *Ppp1r1b*, a dopamine- and cAMP-regulated inhibitor of protein phosphatase-1 (28) (Figures 4E,F).

Western blot analysis revealed increased protein abundance of PEPCK (Figures 4G,H) and CLCNKA (Figures 4I,J) in the HP compared to NP and LP diet groups. Immunofluorescence for SNAT3, the protein encoded by *Slc38a3*, revealed basolateral membrane expression in proximal (LTL-positive) and distal (LTL-negative) tubules of both low and high protein diet fed mice. Additionally, SNAT3 was expressed in dilated tubules in mice fed a HP diet (Figure 4K). Ammonia (NH3), generated from glutamine metabolism, excretes acid into the urine (29). Thus, we examined whether *Pkd1KO* mice retain or excrete more ammonia on a HP diet. Urinalysis revealed increased NH3 levels in HP compared to LP or NP fed mice (Figure 4L), while no differences were observed in kidney tissue (Figure 4M). Collectively, these data suggest that changes in glutamine metabolism and/or ion transport contribute to early hypertrophy and cyst growth induced by a HP diet.

### Glutamine supplementation induces kidney hypertrophy in *Pkd1KO* mice

**Figure 5:**
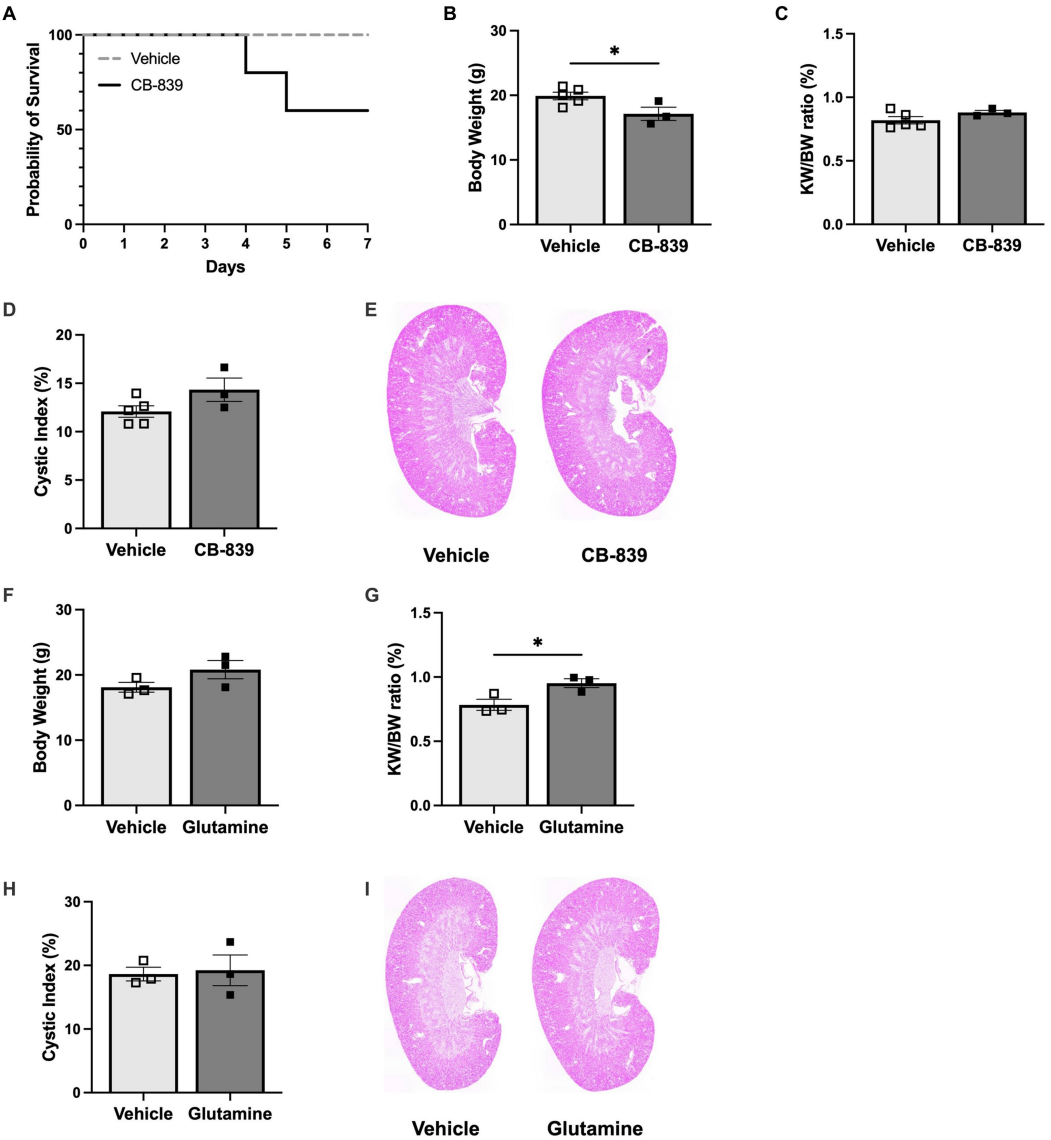
Glutamine stimulates early kidney hypertrophy in *Pkd1KO* mice. **(A)** Daily CB-839 (100 mg/kg, OG) treatment for 1 week with high protein feeding decreased survival compared to vehicle treatment. **(B–E)** Body weight, KW/BW ratio, representative kidney histology and cystic index of mice treated with vehicle or CB-839. With a reduction in BW, no difference was observed in KW/BW or cyst expansion between treatment groups. **(F–I)** BW, KW/BW ratio, representative kidney histology and cystic index of mice treated with vehicle or glutamine (5 g/kg, OG) daily for 1 week with normal protein feeding. KW/BW ratio increased in mice given glutamine, while no differences where observed in cystic index. Results of unpaired, Student’s *t*-test reported. *p < 0.05.

In ADPKD, glucose is disproportionately converted into lactate instead of pyruvate, which is necessary to fuel the tricarboxylic acid (TCA) cycle. This metabolic reprogramming allows glutamine to be used as an alternative energy source to spin the TCA cycle via intracellular conversion to glutamate by glutaminase (30). Therefore, to further delineate the role of glutamine in altering early growth dynamics, *Pkd1KO* mice fed a high protein diet were dosed with vehicle or glutaminase inhibitor CB-839 for 1 week. Treatment with CB-839 appeared to be toxic, as 40% of experimental mice died before the end of the study (Figure 5A). Remaining mice were hunched, weighed significantly less than vehicle treated mice (Figure 5B), and presented with liver discoloration. Consequently, there was no difference in KW/BW ratio (Figure 5C) or cystic index (Figures 5D,E) between vehicle and CB-839 treated mice.

To combat these adverse effects, *Pkd1KO* mice were instead fed a normal protein diet and gavaged with vehicle or glutamine for 1 week. Glutamine supplementation did not alter body weight (Figure 5F), but it did significantly increase kidney growth (Figure 5G) compared to vehicle. There were no differences in cystic index (Figures 5H,I). This implies that glutamine is an essential contributor to early kidney growth observed with HP intake.

## Discussion

Our previous study identified resident MФs as key drivers of accelerated cystogenesis in *Pkd1KO* mice following compensatory renal hypertrophy by unilateral nephrectomy (22). The present study found that dietary protein load results in renal hypertrophy and cyst growth, accompanied by increases in both infiltrating and resident MФs in *Pkd1KO* mice. Suppressing MФs with clodronate treatment mitigates the inflammatory response and slows cystic acceleration induced during HP intake. Moreover, HP diet stimulates alternative energy metabolism within kidney tubular epithelial cells, progressing cyst growth prior to MФ recruitment in early stages of cystic development.

High protein intake increases the delivery of amino acids to the kidney triggering hypertrophy (14, 31). Of note, there were no substantial phenotype differences observed between NP and HP fed *Pkd1KO* mice during the 6 week intervention. However, NP-fed *Pkd1KO* mice had a higher daily food intake compared to HP-fed knockouts and increased caloric intake is known to promote cystogenesis (32, 33). When NP diet was restricted to approximately match the caloric intake to that of the HP and LP groups, it significantly reduced the number of MФs, expression of chemokines and cytokines, and expansion of cysts. Considering mice ate the restricted food within a few hours of replacement, this complies with a fasting period, which is known to slow cystogenesis (34, 35). Whether these changes were due to differences in glucose dynamics or ketosis has yet to be determined.

Previous reports have indicated that cyst progression in ADPKD is largeley shaped by macrophage involvement. Specifically, macrophages were shown to accumulate and home to cystic epithelium contributing to cyst growth in both orthologous (16, 19) and nonorthologous (21) models of PKD, whereas blockade of macrophage colony stimulating factor CSF1 (21) or monocyte chemoattractant protein MCP-1 (19) in these models reduced macrophage numbers and cyst formation. Our data is in agreement with these observations, showing clear macrophage number and cytokine level increases in chronically HP-fed *Pkd1KO* mice that are drastically attenuated by macrophage depletion. Interestingly, this does not appear to be the case in early cyst development and hypertrophy due to protein load in our 1 week model. This is in opposition to Cassini et al. (19), who found highly upregulated *Mcp1* and increased macrophage numbers at a similarly early timepoint soon after induction. However, it is possible that disease progression differs in Cassini’s tubule-specific, TetO-Cre *Pkd1KO* mice versus the global, Cag-Cre *Pkd1KO* mice used in the present study.

Amino acids are metabolized in the mitochondria to contribute to the organelles’ main function of producing energy via oxidative phosphorylation. In ADPKD, this task is known to be impaired, in part, due to the cells inability to fuel the TCA cycle due to impaired glycolysis. This reprogramming, termed the Warburg-like effect, shifts the cell towards aerobic glycolysis, converting pyruvate to lactate instead of entering the TCA cycle, ultimately yielding less adenosine triphosphate (ATP) (30, 36, 37). Due to this lack in energy supply, mitochondria preferentially utilize glutamine to maintain membrane potential and overall function in PKD (38, 39).

Upon initiation of renal hypertrophy and cyst growth induced by a HP diet, we uncovered that kidney epithelial cells increase expression of SNAT3 and PEPCK. SNAT3, a sodium-glutamine transporter localized to the basolateral membrane in proximal tubules, transports glutamine into the cell (26). Glutamine is then converted to glutamate for use in (1) the TCA cycle for gluconeogenesis/ATP synthesis (40) and (2) ammoniagenesis to excrete acid from the kidney (29). This data is supported by previous studies showing increased kidney *Slc38a3* and/or *Pepck1* expression upon HP (41) or high acid diet (41, 42) in wild type mice as well as those suggesting essential metabolic precursors for glucose and ATP metabolism promote PKD (30, 43). Interestingly, SNAT3 was highly expressed in cystic tubular epithelium of our *Pkd1KO* mice fed a NP diet, indicating increased glutamine uptake into cysts. Together, the elevated expression of SNAT3 and PEPCK, which catalyzes gluconeogenesis and mitochondrial oxidative phosphorylation (44, 45), and increased urinary ammonia in HP diet fed mice implies a role for glutamine metabolism early in *Pkd1KO* cyst growth. This study is limited by the absence of available metabolomic profiles for cystic versus noncystic tubular epithelial cells during a high protein diet.

Steidl et al. recently uncovered that glutamine, but not glucose, supplementation is sufficient to produce ATP in cells exposed to metabolic stress, as occurs in ADPKD (46). Therefore, it is unsurprising that inhibiting glutamine intracellular entry would result in weight loss and death in our *Pkd1KO* mice without a viable source of energy. Kidney hypertrophy and cystic index were unchanged by glutaminase inhibition, contrary to studies performed in Aqp2-Cre or HoxB7-Cre Pkd1^fl/fl^ mice (47, 48). These differences may be explained by the fact that both mice are constituitive models that begin to grow kidney cysts *in utero*, whereas our inducible mice begin to form cysts as an adult after Cre induction. Thus, the Aqp2-Cre or HoxB7-Cre mice are likely already in a “proliferative” state at the onset of treatment, unlike CAGG-Cre mice. Lack of cyst growth with CB-839 in our global *Pkd1KO* mice may also be due to inhibiting extrarenal sources of *Pkd1* that could contribute to cyst size, unlike the kidney specific mice. We plan on unraveling these concepts in future studies comparing CAGG-Cre and Ksp-Cre *Pkd1KO* mice. Since loss of body weight with CB-839 made it difficult to discern glutamine’s contribution to early cyst growth from HP diet, extra *Pkd1KO* mice on a NP diet were gavaged with either vehicle or an additional serving of glutamine for 1 week. Glutamine alone was sufficient to induce kidney hypertrophy, emphasizing that, in agreement with Flowers et al. (48), glutamine is essential for early *Pkd1KO* growth.

Another noteworthy finding is that HP diet increased kidney expression of CLCNKA, a transepithelial chloride channel localized to the inner medullary thin ascending limb (tAL) and involved in urine concentrating mechanisms (27). Dehydration upregulates CLCNKA expression in the basolateral membrane of the tAL (49) in addition to the distal tubules (50) to facilitate chloride excretion and sodium reabsorption. Although protein load has not been shown to alter CLCNKA expression in the kidney (51), vasopressin, which is elevated in ADPKD and contributes to cystogenesis (52), has been shown to stimulate kidney CLCNKA expression (53). Whether increased CLCNKA expression from HP diet results in cystogenesis or a urine concentrating defect in ADPKD remains to be determined.

Collectively, this study indicates that dietary protein load in *Pkd1KO* mice increases delivery of glutamine and alternative energy production during early hypertrophy leading to accelerated cyst growth prior to induction of an immune response. In agreement with our unilateral nephrectomy model, HP diet-induced cystogenesis is dependent on macrophage influx and increased inflammation. Future testing of these early metabolic markers will be necessary to decipher the direct contribution of each to cyst expansion.

## Data availability statement

The data presented in the study are deposited in the Gene Expression Omnibus repository, accession number GSE# 225495. Data has been deposited.

## Ethics statement

The animal study was reviewed and approved by University of Alabama at Birmingham IACUC. Written informed consent was obtained from the owners for the participation of their animals in this study.

## Author contributions

RS, SY, JH, and TS conceptualized and designed the study. RS, SY, JH, J-SH, CL, MK, and SS performed experiments. RS and TS drafted the manuscript, interpreted results and prepared figures. RS, SY, JH, CL, DC, and TS analyzed data. All authors contributed to the article and approved the submitted version.

## Funding

This study was supported by a NIH NIDDK Postdoctoral Training Grant (T32 DK007545) to RS and NIH NIDDK grants R03 DK119717 and R01 DK132028 to TS.

## Conflict of interest

The authors declare that the research was conducted in the absence of any commercial or financial relationships that could be construed as a potential conflict of interest.

## Publisher’s note

All claims expressed in this article are solely those of the authors and do not necessarily represent those of their affiliated organizations, or those of the publisher, the editors and the reviewers. Any product that may be evaluated in this article, or claim that may be made by its manufacturer, is not guaranteed or endorsed by the publisher.
