# Supplementary material for: Accelerated cystogenesis by dietary protein load is dependent on, but not initiated by kidney macrophages
Source: Front Med (Lausanne). 2023 Jul 19;10:1173674. doi: 10.3389/fmed.2023.1173674 (PMC10394241; doi:10.3389/fmed.2023.1173674)
Supplement: Supplementary file 1 [file Table_1.DOCX]

**SUPPLEMENTARY**

**Table 1.** *Nutritional composition (g/Kg) per diet*

|  | Low (6%) Protein Diet | Normal (18%) Protein Diet | High (60%) Protein Diet |
| --- | --- | --- | --- |
| Calcium Carbonate | 1.6 | 4.6 | 15.4 |
| Calcium Phosphate, dibasic | 21.6 | 17.36 | 2.35 |
| Casein | 69 | 207 | 695 |
| Cellulose | 57.82 | 41.06 | 20 |
| Corn Oil | 53.9 | 52.6 | 48 |
| Corn Starch | 200 | 200 | 0 |
| DL-Methionine | 0.9 | 2.7 | 0 |
| Ethoxyquin, antioxidant | 0.01 | 0.01 | 0.01 |
| Maltodextrin | 0 | 0 | 55 |
| Mineral Mix | 13.37 | 13.37 | 13.37 |
| Sucrose | 572 | 451 | 141 |
| Vitamin Mix | 10 | 10 | 10 |

**Table 2.** *Amino acid composition (g/Kg) per diet*

|  | Low (6%) Protein Diet | Normal (18%) Protein Diet | High (60%) Protein Diet |
| --- | --- | --- | --- |
| Ala | 1.794 | 5.382 | 18.07 |
| Arg | 2.277 | 6.831 | 22.935 |
| Asp | 4.14 | 12.42 | 41.7 |
| Cys | 0.1725 | 0.5175 | 1.7375 |
| Glu | 12.558 | 37.674 | 126.49 |
| Gly | 1.104 | 3.312 | 11.12 |
| His | 1.725 | 5.175 | 17.375 |
| Ile | 3.45 | 10.35 | 34.75 |
| Leu | 5.52 | 16.56 | 55.6 |
| Lys | 4.83 | 14.49 | 48.65 |
| Met | 2.487 | 7.461 | 15.985 |
| Phe | 3.036 | 9.108 | 30.58 |
| Pro | 6.21 | 18.63 | 62.55 |
| Ser | 3.45 | 10.35 | 34.75 |
| Thr | 2.622 | 7.866 | 26.41 |
| Trp | 0.69 | 2.07 | 6.95 |
| Tyr | 3.174 | 9.522 | 31.97 |
| Val | 4.14 | 12.42 | 41.7 |

**Table 3.** *Primary* *antibodies* *for specified assays*

| **Target** | **Catalog #** | **Company** | **Dilution** |
| --- | --- | --- | --- |
| *Flow Cytometry* | | | |
| CD45, PE | 12-0451-82 | Invitrogen | 1:300 |
| F4/80, eFLUOR 450 | 48-0451-82 | Invitrogen | 1:200 |
| CD11b, APC | 17-0451-82 | Invitrogen | 1:200 |
| Ly-6C, APC-Cy7 | 557661 | BD Biosciences | 1:100 |
| CD11c, PE-Cy7 | 25-0114-82 | Invitrogen | 1:300 |
| Ly6c, PerCP5.5 | 560525 | BD Biosciences | 1:200 |
| CD3e, FITC | 553062 | BD Biosciences | 1:100 |
| I-A/I-E, BV650 | 107641 | Biolegend | 1:200 |
| LIVE/DEAD | L34966 | Invitrogen | 1:500 |
| *Western Blot* | | | |
| PEPCK | 10004943 | Cayman Chemical | 1:500 |
| CLCNKA | mbs129305 | MyBioSource.com | 1:1000 |
| β-Actin | AM4302 | Invitrogen | 1:10000 |
| *Immunofluorescence* | | | |
| SNAT3 | sc-398982 | Santa Cruz Biotechnology, Inc. | 1:50 |

**Table 4.** *Primers* *for quantitative RT-PCR*

| **Gene** | **Direction** | **Sequence (5'→3')** |
| --- | --- | --- |
| *Il6* | Forward | TAGTCCTTCCTACCCCAATTTCC |
|  | Reverse | TTGGTCCTTAGCCACTCCTTC |
| *Ccl2* | Forward | TGATCCCAATGAGTAGGCTGGAG |
|  | Reverse | ATGTCTGGACCCATTCCTTCTTG |
| *Tgfb1* | Forward | CTTCAATACGTCAGACATTCGGG |
|  | Reverse | GTAACGCCAGGAATTGTTGCTA |
| *Havcr1* | Forward | AGCAGTCGGTACAACTTAAAGG |
|  | Reverse | AGAGTTCTCTATCGTCAAGGACA |
| *Cmyc* | Forward | CCCTATTTCATCTGCGACGAG |
|  | Reverse | GAGAAGGACGTAGCGACCG |
| *Pepck1* | Forward | CCACAGCTGCTGCAGAACA |
|  | Reverse | GAAGGGTCGCATGGCAAA |
| *Slc38a3* | Forward | CTCTTCGGCTACCTCACCTTCT |
|  | Reverse | AAGTGTGACCGCTATCAGCACG |
| *Clcnka* | Forward | TGCCATGAACTTTGCTATCGG |
|  | Reverse | GAGAAGGGCGTAATGCTCTGT |
| *Ppp1r1b* | Forward | ACCCCTGCCATGCTTTTCC |
|  | Reverse | TTGGGTCTCTTCGACTTTGGG |
| *Gapdh* | Forward | AGGTCGGTGTGAACGGATTTG |
|  | Reverse | TGTAGACCATGTAGTTGAGGTCA |

**Supplemental Figure 1.** **Average body weight and food intake during 6 week dietary intervention.** (**A**) Average body weight. *Pkd1KO* mice fed a Normal-RF diet weighed less than those consuming ad libitum diets. Results of two-way ANOVA with Tukey’s multiple comparisons post hoc test showed no significant differences between flox and KO mice. (**B**) Daily food intake of *Pkd1KO* mice normalized to average BW. Despite restricting dietary intake, mice fed a Normal-RF diet had similar food intake to NP ad libitum. Results of an Ordinary one-way ANOVA with Tukey’s multiple comparisons test amongst diet groups in *Pkd1KO* mice is reported. *P* **< 0.01, ***< 0.001.

**Supplemental Figure 2.** **MA plots of differentially expressed genes between renal epithelial cells from HP versus NP or LP diet fed *Pkd1KO* mice.** (**A**) Genes with upregulated expression in HP compared to NP cells. (**B**) Genes with upregulated expression in HP compared to LP cells. Red arrows denote upregulated genes while green arrows denote downregulated genes.
