# Supplementary figures and images for: Accelerated cystogenesis by dietary protein load is dependent on, but not initiated by kidney macrophages
Source: Front Med (Lausanne). 2023 Jul 19;10:1173674. doi: 10.3389/fmed.2023.1173674 (PMC10394241; doi:10.3389/fmed.2023.1173674)

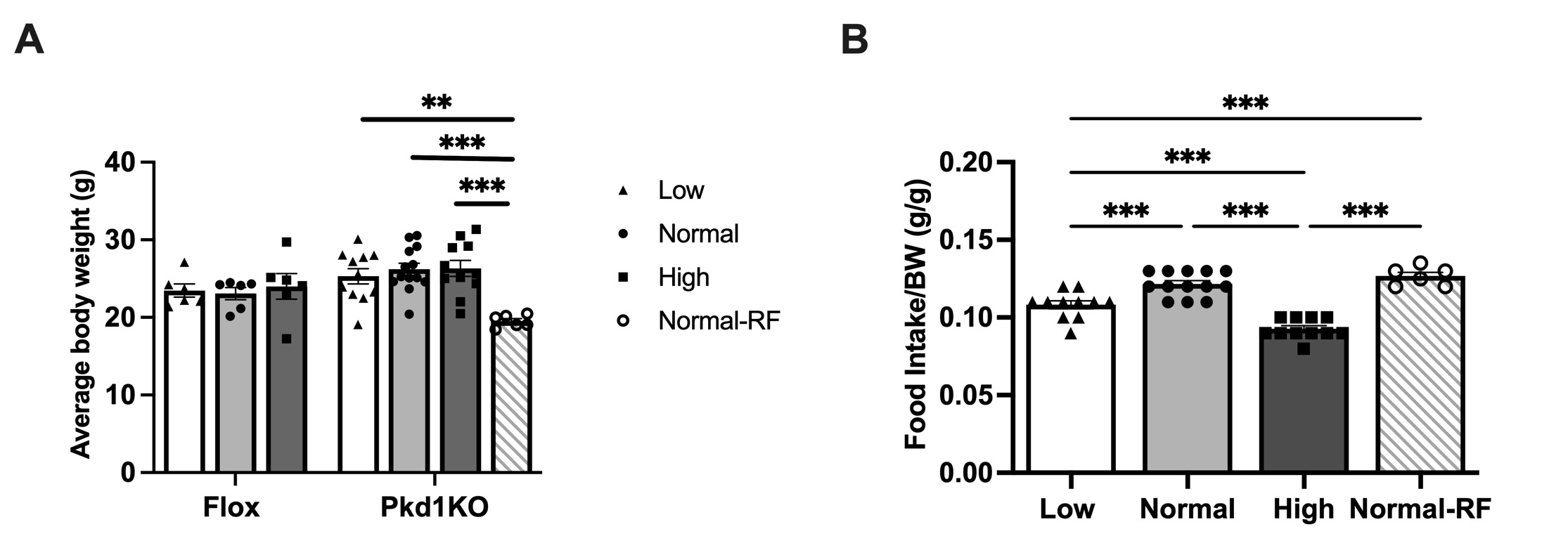

Supplement: Supplementary file 2 [file Image_1.JPEG]

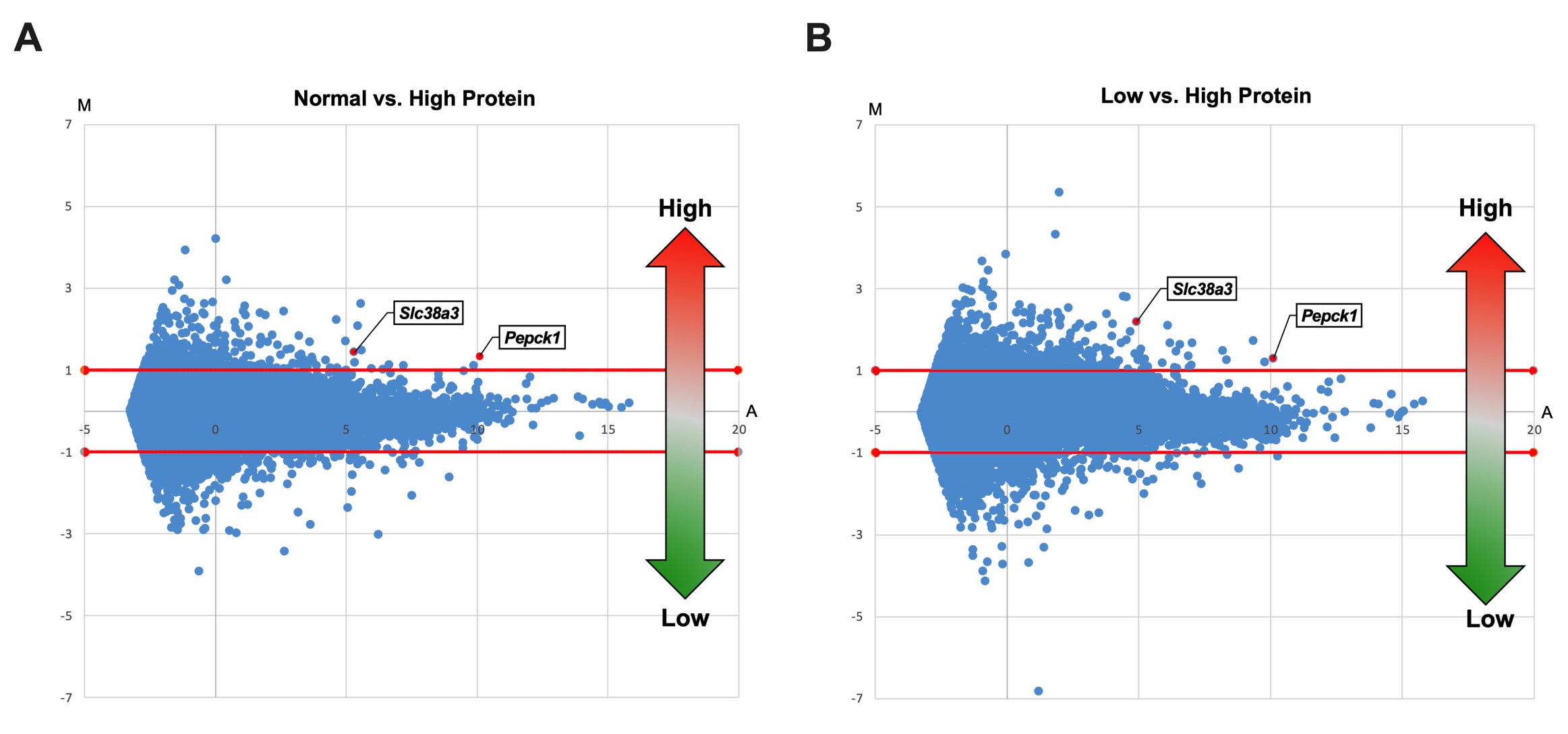

Supplement: Supplementary file 3 [file Image_2.JPEG]
